# Patterns of recurrence and outcomes of glioblastoma multiforme treated with chemoradiation and adjuvant temozolomide

**DOI:** 10.6061/clinics/2020/e1553

**Published:** 2020-09-03

**Authors:** Alexandre Ciuffi Faustino, Gustavo Arruda Viani, Ana Carolina Hamamura

**Affiliations:** Faculdade de Medicina de Ribeirao Preto (FMRP), Universidade de Sao Paulo, Ribeirao Preto, SP, BR.

**Keywords:** Glioblastoma multiforme, Radiotherapy, Chemotherapy, Survival

## Abstract

**OBJECTIVES:**

To assess the patterns of failure and prognostic factors in Brazilian patients with glioblastoma multiforme (GBM) treated with radiotherapy (RT) and concurrent and adjuvant temozolomide (TMZ).

**METHODS:**

Patients with diagnosed GBM post-resection received postoperative RT. TMZ was administered concurrently at 75 mg/m^2^/day for 28 consecutive days and adjuvant therapy at 150-200 mg/m^2^/day for 5 days every 28 days. Radiographic failure was defined as any new T1-enhancing lesion or biopsy-confirmed progressive enhancement inside of the radiation field. When possible, patients with recurrence were salvaged with metronomic TMZ, either in combination with a local treatment or alone (surgery or re-irradiation). Several prognostic factors were evaluated for overall survival (OS). Univariate and multivariate analyses were performed to identify significant factors. A *p*-value <0.05 was considered significant.

**RESULTS:**

This study included 50 patients. The median follow-up time was 21 months. The median RT dose was 60 Gy and all patients received concomitant TMZ. During follow-up, 41 (83.6%) failures were observed, including 34 (83%) in-field, 4 (9.7%) marginal, and 3 (7.3%) distant failures. Metronomic TMZ was used as salvage treatment in 22 (44%) cases and in combination with local treatment in 12 (24%) cases. The median OS and progression-free survival times for the entire cohort were 17 and 9 months, respectively. In univariate analysis, the following factors were significant for better OS: maximal surgical resection (*p*=0.03), Karnofsky Performance Score (KPS)>70 at diagnosis (*p*=0.01), metronomic TMZ treatment (*p*=0.038), recursive partitioning analysis class III (*p*=0.03), and time to failure >9 months (*p*=0.0001). In multivariate analysis, the following factors remained significant for better OS: metronomic TMZ (*p*=0.01) and time to failure >9 months (*p*=0.0001).

**CONCLUSION:**

The median OS of Brazilian patients with GBM treated with RT and TMZ was satisfactory. Although TMZ therapy has become the standard of care for patients with newly diagnosed GBM, the recurrence rate is extremely high. Metronomic TMZ as salvage treatment improved survival in these patients.

## INTRODUCTION

Although high-grade gliomas are relatively rare tumors in adults, they are the most common primary tumors from the brain, accounting for 2% of all cancer cases in adults (1). High-grade gliomas are generally classified as anaplastic astrocytomas, anaplastic oligodendrogliomas, and glioblastoma multiforme (GBM) (2). GBM is the most common high-grade glioma and is considered a lethal disease affecting young adults (1). The treatment outcomes of patients with GBM are better with multidisciplinary team approaches than with isolated modalities (3). Unfortunately, despite the combination of modalities including surgery, radiotherapy (RT), and chemotherapy, the survival of these tumors remains poor (3-9). Currently, the standard treatment involves maximal surgical resection followed by concomitant chemoradiation (3). Multimodal treatment was established as the standard for GBM in 2005, after the publication of a remarkable randomized clinical trial (3). In this trial, 573 patients were randomized to postoperative RT (60 Gy) combined with temozolomide (TMZ, 75 mg/m^2^) during RT, followed by six cycles of adjuvant TMZ (150-200 mg/m^2^ for 5 days each 28 days) *versus* the same RT schedule without chemotherapy. The combined treatment achieved a significant difference in 1-year survival (27% *versus* 11%) (3). In Brazil, until 2011, most public institutions did not administer TMZ to patients in the Brazilian health care system. Thus, data on the treatment outcomes and patterns of relapse/progression of GBM in Brazilian patient cohorts are scarce.

This study aimed to show the pattern of relapse, prognostic factors, and outcomes of Brazilian patients with GBM treated with RT in combination with TMZ in a tertiary public institution.

## MATERIALS AND METHODS

This retrospective cohort study was performed between 2009 and 2017 at the Radiation Oncology Department of a tertiary public center. The local ethics committees approved the study.

### Inclusion criteria

Patients with a diagnosis of GBM by biopsy and brain magnetic resonance imaging (MRI) treated with postoperative RT and TMZ were selected for the study. Regarding RT techniques and doses, three-dimensional conformal radiation therapy (3D-CRT) or intensity-modulated radiotherapy (IMRT) with a total dose ≥54 Gy, using merge image techniques (MRI with computerized tomography [CT]), were included. Any degree of surgical resection (only biopsy, partial, or complete) was permitted. The chemotherapy schedule consisted of TMZ combined with RT and included only patients who received all the planned doses of TMZ during RT and at least one cycle of adjuvant TMZ after RT.

### Exclusion criteria

Patients without a history or physical examination, without imaging studies before surgery, with a diagnosis of low-grade glioma, or with previous radiotherapy treatment in the brain were excluded from this analysis. Patients with inadequate doses (<54 Gy) without adjuvant treatment with TMZ or with an inadequate follow-up were also excluded.

### Treatment

3D-CRT or IMRT were performed with a linear accelerator with an energy of 6 MV. All patients underwent CT scan simulation for treatment planning. The patients were placed in dorsal decubitus with a thermoplastic mask to guarantee adequate immobilization during treatment.

Three-millimeter-thick CT slices were acquired from the top of the skull to the larynx. The following structures were delineated from the collected images: brain stem, optic chiasm, optic nerves, lenses, retinae, cochleae, and temporal lobes. To determine the tumor volume to be treated, postoperative brain MRI was merged with the planning CT. The guidelines from the Radiation Therapy Oncology Group (RTOG) and European Organization for Research and Treatment of Cancer (EORTC) (4,5) were used to draw the clinical target volume and planning treatment volume. According to the RTOG, two volumes were generated. In the first treatment volume, using T2-weighted brain MRI, the tumor plus edema were delineated and expanded with 2 cm margins in all directions. For this treatment volume, a total dose of 44 Gy in 22 fractions was delivered. After that, using T1-weighted MRI, the tumor was delineated and expanded by 1 cm in all directions. For this second volume, a total dose of 60 Gy in 30 fractions was delivered. Consistent with EORTC, using T1-weighted MRI, the tumor or tumor cavity was delineated and expanded by 1-2 cm in all directions. In CT planning, after determining the treatment volumes, the treatment fields were chosen and the doses to the organs at risk (OAR) were checked. The dose limitations to the OAR followed the Quantitative Analysis of Normal Tissue Effects in the Clinic recommendations as follows: brainstem, 54 Gy; spinal cord, 45 Gy; chiasm, 50-54 Gy; retinae, 45 Gy; and lenses, 10 Gy. TMZ was administered concurrently at 75 mg/m^2^/day for 28 consecutive days and adjuvant at 150-200 mg/m^2^/day for 5 days every 28 days.

### Data collection

A structured table was created to collect data about age, sex, comorbidities, tumor location, neurological symptoms, medication use, clinical performance, total RT dose, RT, recursive partitioning analysis (RPA) class, tumor location, surgery extension, and chemotherapy for all patients. During RT, patients were checked for the presence of adverse effects such as headaches, convulsion, motor deficits, sensitive deficits, nausea, and vomiting. The adverse effects were graduated according to the Common Toxicity Criteria (CTC), version 3.0. Data on toxicity equal to or higher than grade II were collected. After treatment, patients were followed up every 3-4 months in the first year and 4-6 months thereafter.

### Recurrence/progression and salvage treatment

Patients with disease progression or recurrence during follow-up were evaluated by the multidisciplinary team to discuss the salvage treatment options. In general, recurrences located in non-eloquent areas in patients with good clinical performance were salvaged either by surgical resection alone or in combination with chemotherapy. In cases where a surgical resection was not possible, local re-irradiation was considered. For recurrence or progressive cases with no indication or clinical condition to the local treatment, metronomic TMZ was considered depending on the patients clinical performance. Patients unable to receive local salvage treatment (surgery or re-irradiation) were administered metronomic TMZ alone at a 40-50 mg/m^2^/day daily until disease progression.

### Statistical analyses

For continuous variables, means and standard deviation were calculated. Dichotomous variables were treated as proportions and percentages. Time to death and time to recurrence were counted in months from diagnosis by biopsy. Radiographic failure was defined as any new T1-enhancing lesion or biopsy-confirmed progressive enhancement at the primary site. MRIs obtained at the time of failure were fused to the original RT plans. In-field, marginal, and distant failures were designated as recurrence inside the 95% isodose, recurrence between 95% and 20%, and recurrence outside the 20% isodose of the prescribed dose, respectively. Several prognostic factors were evaluated for overall survival (OS). Univariate and multivariate analyses were performed to identify significant factors. *p*-values <0.05 were considered significant and variables significant in univariate analyses (*p*<0.05) were included in the multivariate analysis by the Cox regression method. Statistical analysis was conducted using SPSS version 20.0.

## RESULTS

During the study period, 50 patients fulfilled the inclusion criteria and were included in the cohort. Most of the patients were male (60%), with a mean age of 54 (±11.5y) years of age. Maximal surgical resection was performed in 30 (60%) patients. The median RT dose was 60 Gy (range 54-60 Gy), with all patients receiving concomitant TMZ. IMRT and 3D-CRT were the radiation techniques employed in 28 (57%) and 22 (43%) of patients, respectively. According to RPA prognostic classification, RPA class IV was the most common (80%, Table 1). The planned RT+TMZ was administered in 88% of patients, with 60% of patients receiving six adjuvant cycles. The causes for interruption of adjuvant treatment were disease progression (24% of cases), treatment toxicity (6%), and other (10%) (Table 4).

**Figure 1:**
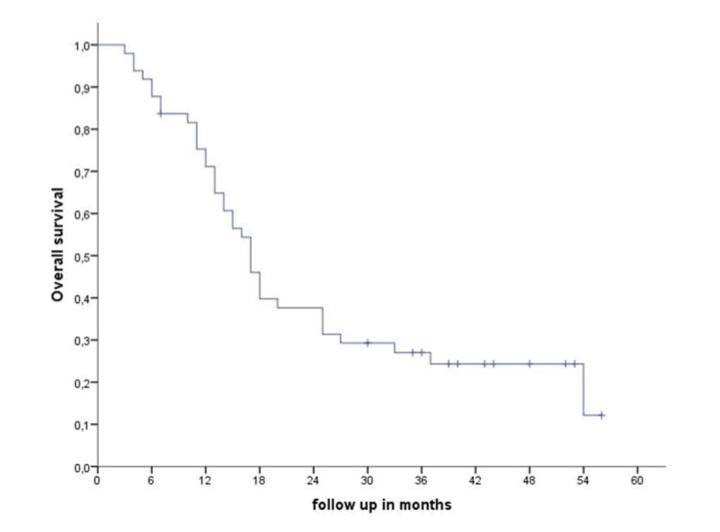
Overall survival for glioblastoma multiforme treated with adjuvant chemoradiation.

**Figure 2:**
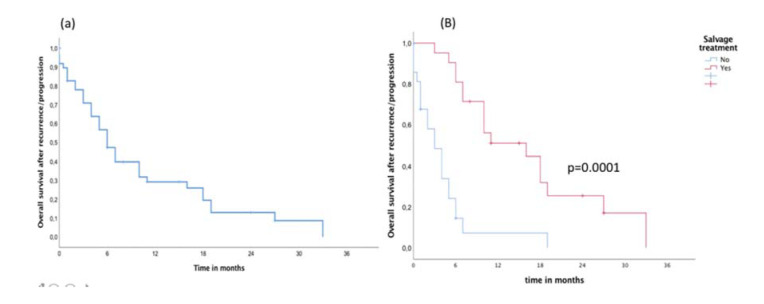
(A) Overall survival post recurrence/progression independent of salvage treatment and (B) Overall survival post recurrence/progression in patients with and without salvage treatment.

In the entire cohort, the median follow-up time was 22 months. During follow-up, a total of 41 (82%) failures were detected, including 34 (83%) in-field, 4 (9.7%) marginal, and 3 (7.3%) brain distant failures. Metronomic TMZ was used as salvage treatment in 22 (44%) patients and was combined with local treatment (surgery or re-irradiation) in 12 (24%) patients. Surgical resection as a local treatment for salvage was possible in eight (66%) patients and re-irradiation was administered to four (34%) patients. The median OS and failure-free survival times of the entire cohort were 17 and 9 months, respectively. During the follow-up, 38 (76%) patients died from the disease, with only 12 patients alive (Figure 1). After recurrence/progression, the OS independent of the salvage treatment was 10 months (95% confidence interval [CI] 7-13 months) (Figure 2A). The median OS after recurrence/progression differed significantly between patients with (16 months, 95%CI 11-20 months) and without salvage treatment (4 months, 95%CI 2-6 months) (*p*=0.0001, Figure 2B).

The univariate analysis revealed the following factors to be significantly associated with better OS: maximal surgical resection (*p*=0.03), Karnofsky Performance Score (KPS) >70 for diagnosis (*p*=0.01), metronomic TMZ salvage treatment (*p*=0.038), RPA-III (*p*=0.03), and time to failure >9 months (0.001) (Table 2). In the multivariate analysis, the following factors remained significantly associated with better OS: metronomic TMZ (*p*=0.04) and time to failure >9 months (*p*=0.002) (Table 3). Regarding treatment toxicity, according to the CTC version 3.0, the most common side effects were grade 2 thrombocytopenia (12% of patients), anemia (6%), and neutropenia (5%). Three patients (6%) developed grade 3 neutropenia.

## DISCUSSION

GBM accounts for approximately 60% of all primary malignant tumors of the brain (1). The therapeutic results for any treatment modality (surgery, RT, or chemotherapy) are dismal. Over the last few years, many efforts have been made to improve the survival of this aggressive and fatal disease. In 2005, a randomized clinical trial showed significantly better 2- and 5-year survival for patients administered combined chemoradiation (3). However, in Brazil, this treatment was not covered by the Brazilian public health system until the end of 2011.

To our knowledge, this study is the first to report the outcomes of Brazilian patients with GBM treated with chemoradiation. Our data confirm the high rate of treatment compliance (88%) with a low rate of severe hematological toxicity and a reduced rate of treatment interruption, which contributed to a satisfactory rate of cycles completed using combined treatment in this population. This data reinforces the clinical applicability of the combined treatment proposed by Stupp et al. in Brazilian patients, and shows that it is possible to achieve similar outcomes in treating this aggressive disease outside of a clinical trial.

GBM is a resistant tumor with a high incidence of brain recurrence or disease progression even after multimodality treatment (2).

The treatment policy of following-up patients with GBM with brain MRI to detect early brain failure and selecting patients at brain recurrence or progression to receive salvage treatment with metronomic TMZ, whether or not in combination with a local treatment, yielded a median OS of 17 months. In general, local treatment for salvage was possible in 12 patients, with only eight patients with clinical conditions or tumors located in non-eloquent areas able to undergo surgical resection. Twenty-eight patients had a poor clinical condition (KPS <70) at the recurrence/progression and were administered metronomic TMZ.

These data called our attention to possible significant prognostic factors related to survival. In our analysis, several prognostic factors were significant. Among them, metronomic TMZ (*p*=0.04) and time to failure >9 months (*p*=0.002) remained significant in multivariate analysis.

Although we did not find a significant correlation between the time to failure and maximal surgical resection, most patients with a long time to failure received maximal surgical resection. Murakami et al. evaluated the significance of the extent of surgical resection and the progression-free interval (10). They observed 3, 4, and 8 months of progression-free survival, respectively, for biopsied tumors, tumors with partial resection, and maximal resection (*p*<0.05). Methylation of the promoter for O6-methylguanine-DNA methyltransferase (MGMT) is a significant prognostic factor related to better OS and can predict the benefit from chemotherapy (6). For instance, in a later publication of the randomized trial conducted by the NCI/EORTC, Stupp et al. identified that patients with GBM and MGMT methylation had 2-year OS rates of 49% and 24% with combination therapy and RT alone, respectively, compared to 15% and 2%, respectively, for those without MGMT methylation (6). Unfortunately, in our analysis, we could not evaluate the MGMT status of our patients; thus, some patients with a longer time to failure and better OS may have MGMT methylation.

After RT treatment, up to 80% of patients experience recurrence inside of the RT field (11); in this setting, there is currently no consensus regarding the best option for salvage treatment (12). Several studies have described the outcomes of different salvage treatments in progressive or recurrent GBM (12-15). A phase II study including 35 cases with recurrent GBM tested the combination of bevacizumab and irinotecan. The 6-month PFS was 46%, with a partial response rate of 57% (15). However, in Brazil, combined treatment with bevacizumab is not covered by the public health system. Continuous administration of specific cytotoxic agents at low doses, known as “metronomic chemotherapy,” has been reported to be more efficient in targeting the GBM tumor endothelium. The theoretical advantage of metronomic chemotherapy is that it does not allow GBM-associated endothelial cells to recover. A phase II trial assessed metronomic TMZ as a salvage treatment in GBM (13). Metronomic TMZ resulted in 6-month PFS and OS rates of 32% and 56%, respectively (13). Another positive point for using metronomic TMZ as salvage treatment is its effectiveness against GBM independent of MGMT status by inhibiting tumor endothelial proliferation (16-18).

Therefore, in our institution, metronomic TMZ has been employed as a salvage therapy in this clinical scenario, either alone or in combination with a local treatment. The role of local therapy is also not well established, being reserved for patients with significant neurological symptoms when the disease and patients are operable (12). In the last decade, the use of re-irradiation has significantly increased mainly because of improvements in the accuracy of image-guided radiotherapy and knowledge regarding radiation tolerability by the brain (19). Several radiation schedules have been used with total doses ranging from 24-36 Gy in 4-6 fractions (16). In well-selected patients, re-irradiation produces progression-free OS rates comparable to surgery and chemotherapy. In our data, most of the brain recurrence was in-field and inside of the 95% isodose curve. This clinical situation considerably limits salvage treatment with a new course of radiotherapy or further surgical resection. Based on this pattern of recurrence, only 24% of patients from our cohort received local salvage treatment combined with metronomic TMZ.

Nevertheless, the tumor location concerning the isodose curve is not the only factor influencing the decision regarding salvage treatment (12). Many other variables must be considered in the decision making for salvage treatment, such as clinical performance, tumor size, age, professional experience, and patient preference (12). The KPS at recurrence is a crucial variable to determine if the patient is capable of tolerating salvage treatment. In our analysis, a KPS above 60 was not significantly associated with OS. However, KPS classification is subjective, variable over time, and can induce errors.

Consequently, we have used the KPS only to select patients for more intensive treatment. Patients with higher KPS (90-100) at recurrence with favorable tumor locations were selected for local therapy and metronomic TMZ; thus, the KPS was not statistically significant in univariate analysis. In contrast, patients with lower KPS (60-80) at recurrence received only metronomic TMZ treatment and patients with KPS <50 were considered for best supportive care. In general, patients undergoing salvage treatment had a median survival of 20 months compared to 13 months in patients without salvage treatment. Regarding the type of salvage treatment, no significant difference was observed between TMZ with or without local treatment.

## CONCLUSION

The survival achieved in a cohort of Brazilian GBM patients treated by RT combined with TMZ was similar to that observed in a randomized clinical trial. This finding shows that it is possible to achieve similar outcomes, even in the treatment of an aggressive and fatal disease, outside of a clinical trial.

The combined treatment was well-tolerated, with a lower rate of treatment interruption and satisfactory adherence. The recurrences were preponderantly in-field when local treatment was challenging, and limited to patients with a favorable tumor location and excellent clinical performance. In this clinical scenario, metronomic TMZ, whether or not in combination with a local treatment, produced better survival. The accessibility of TMZ in the Brazilian public health system makes metronomic TMZ in patients with GBM recurrence an adequate treatment choice. The critical prognostic factors for survival found here might be useful for the management of patients with GBM in clinical practice.

## AUTHOR CONTRIBUTIONS

Faustino AC was responsible for the data collection, file review and manuscript writing. Viani GA was responsible for the statistical analyses, manuscript review and supervision. Hamamura AC was responsible for the data collection.

## Figures and Tables

**Table 1:** Demographic characteristics of patients with glioblastoma.

Variables	Number (%)
Sex	
Male	30 (60)
Female	20 (40)
Age (years)	54 (range 36-78)
KPS	80 (range 50-100)
Histology	
Glioblastoma multiforme	50 (100)
Symptoms	
Headache	26 (74)
Convulsion	14 (40)
Nausea/vomiting	22 (63)
Motor deficit	11 (31)
Sensitive deficit	8 (23)
Comorbidities	
Hypertension	12 (25)
Diabetes	3 (6)
Smoking	9 (17)
Surgery	
Total	30 (60)
Partial	16 (32)
Biopsy	4 (8)
Medication	
Dexamethasone	36 (72)
Anticonvulsivant	28 (55)
Radiotherapy	
Total dose	60 Gy/30 fractions (range 54-60)
FLAIR volume (cm^3^)	
RPA classification	
III	8 (15)
IV	39 (80)
V	3 (5)
TMZ+RT	50 (100)
TMZ+RT as planned TMZ+RT interrupted/reduced	44 (88) 6 (12)
Median cycles of adjuvant TMZ	5 (range 1-7)
Follow-up (months)	22 (range 6-50)

**Table 2:** Univariate analysis for overall survival in patients with glioblastoma multiforme.

Variables	2-year OS c (%)	*p*-value
Age (years)		
≥65	23	0.920
<65	30	
Maximal resection		0.037
Yes	34	
No	15	
KPS at diagnosis		0.014
KPS ≥70	26	
KPS <70	14	
RPA class		0.039
III	57	
IV-V	19	
Interval time to failure (months)		0.0001
≥9	45	
<9	8	
KPS at recurrence		0.300
≥60	25	
<60	20	
Salvage with metronomic TMZ		0.009
Yes	33	
No	17	

**Table 3:** Multivariate analysis for overall survival in patients with glioblastoma multiforme.

Variables	Hazard Risk	95%CI	*p*-value
Maximal surgical resection			0.158
No	1	0.8-3.2	
Yes	1.64		
KPS at diagnosis			0.269
KPS ≤70	1	0.6-5	
KPS >70	1.86		
Metronomic TMZ			0.001
No	1	2.1-10.8	
Yes	4.5		
Time to failure (months)			0.0001
<9	1		
≥9	4.6	2-10	
RPA class			0.102
IV/V	1	0.9-10	
III	2.9		

**Table 4:** Characteristics- of this cohort and a randomized clinical trial with glioblastoma multiforme treated with concomitant temozolomide and radiotherapy followed by adjuvant temozolomide.

Variables	NCIC EORTC	Current study
Age (years)	56	54
Range	19-70	36-78
KPS Range	-	80 50-100
Sex		
Male	185 (64%)	30 (60%)
Female	102 (36%)	20 (40%)
Surgery		
Maximal	113 (39%)	30 (60%)
Partial	126 (44%)	16 (32%)
Biopsy	49 (175)	4 (8%)
Median survival	14.6	17
Median progression-free survival	6.9	9
2-year overall survival	26.5%	37.7%
